# In Captive Rhesus Macaques, Cervicovaginal Inflammation Is Common but Not Associated with the Stable Polymicrobial Microbiome

**DOI:** 10.1371/journal.pone.0052992

**Published:** 2012-12-28

**Authors:** Gregory Spear, Kristina Rothaeulser, Linda Fritts, Patrick M. Gillevet, Christopher J. Miller

**Affiliations:** 1 Department Immunology/Microbiology, Rush University Medical Center, Chicago, Illinois, United States of America; 2 Center for Comparative Medicine, University of California Davis, Davis, California, United States of America; 3 California National Primate Research Center, University of California Davis, Davis, California, United States of America; 4 Microbiome Analysis Center, George Mason University, Manassas, Virginia, United States of America; 5 Department of Environmental Science and Policy, George Mason University, Manassas, Virginia, United States of America; Emory University School of Medicine, United States of America

## Abstract

Vaginal inoculation of rhesus macaques (RM) with simian immunodeficiency virus (SIV) has been used to study the biology of HIV transmission. Although the results of vaginal SIV transmission experiments could be affected by vaginal inflammation, studies to date have been conducted without regard to levels of pre-existing genital inflammation present in RM. We collected cevicovaginal secretions (CVS) from 33–36 RM during the mid menstrual cycle (day 10–20) at 2 time points approximately 8 months apart and characterized the mRNA and protein levels of inflammatory cytokines, chemokines and interferon-stimulated genes. There was extreme variability in the levels of inflammatory mediators (IFN-α, IFN-γ, IL-6, TNF, IL-1b, IP-10, MIG, IL-12 and IL-17). In most animals, the mRNA levels of the inflammatory mediators were similar in the 2 CVS samples collected 8 months apart, suggesting that genital inflammation is stable in a subset of captive female RM. At both time points the cervicovaginal microbiota had low levels of *Lactobacillus* and was relatively diverse with an average of 13 genera in the samples from the first time point (median 13, range 7–21) and an average of 11.5 genera in the samples from the second time point (median 11, range 5–20). Many of the macaques had similar microbiota in the samples collected 8 months apart. However, we found no correlation between specific bacterial genera and the mRNA or protein levels of the inflammatory mediators in the genital tract of RM in this study. It seems likely that results of published vaginal SIV transmission experiments in RM have been influenced by pre-existing inflammation in the animals used for the experiments.

## Introduction

Most HIV-1 infections occur by sexual transmission and the presence or absence of genital inflammation is of fundamental importance in HIV transmission [1] since epidemiologic studies suggest that HIV-1 acquisition is increased in women with bacterial vaginosis (BV) or sexually transmitted infections (STIs), especially herpes simplex virus type 2 (HSV-2) [2]–[8]. In women, the genital microbiota influences the expression of proinflammatory cytokines [9], [10]. A consistent finding is that levels of IL-1b are increased in women with bacterial vaginosis compared to women with a genital microbiota that is dominated by *Lactobacillus*. Some studies also show increased levels of IL-8, IL-1α, IL-6, TNF-α and RANTES although these increases are not found in all studies. The genital microbiota also affects susceptibility of women to HIV heterosexual transmission, as HIV acquisition is enhanced by the presence BV [3], [4], [6], [10]–[12]. BV associated inflammation is initiated by the innate immune system after bacterial products bind Toll-like receptors [11], [13]. Ligand binding to the TLRs results in signaling through MYD88 or TRIF that in turn activates the rapid acting transcription factor, NF-kB. Activated NFkB drives transcription of cytokine and adhesion molecule genes, dramatically enhancing expression levels and activating T cells. The infiltrates of activated CCR5+ CD4+ T cells and dendritic cells in the genital mucosa of women with BV and HSV-2 [14]–[20] provide more target cells for HIV infection.

The SIV/rhesus macaque system is a well-developed animal model that can be used for understanding the effects of genital inflammation on HIV transmission. In this animal model the phenotypic and genotypic nature of the virus inoculum is defined, the timing of the virus exposures is known and the genetics (MHC-1 haplotype, TRIM5a polymorphisms) of the animals can be defined. The vaginal microbiota of 2 populations of captive macaques was described in recent NexGen microbiome studies and, compared to humans, macaques have a relatively diverse microbiome although the most prevalent genera are those found in humans with BV [21], [22]. As vaginal transmission experiments in rhesus macaques could be affected by this BV-like flora, we investigated the relationship between the vaginal microbiota and the levels of several soluble proinflammatory mediators in rhesus macaques (RM).

Based on mRNA and protein levels of proinflammatory cytokines and chemokines in cervicovaginal secretions (CVS), we found that the degree of cervicovaginal inflammation in captive RM spans a broad range from minimal to severe. Further we found that the level of genital inflammation, as judged by mRNA levels of cytokines in CVS, in individual animals was relatively stable in 2 samples collected 8-months apart. In an effort to explain this inflammation, we characterized the vaginal microbiome of the animals and found that the microbiota was relatively diverse and *Lactobacillus* was relatively rare. Many of the macaques had similar microbiome patterns at the two time points, examined. However, we found no correlation between specific bacterial genera and the mRNA or protein levels of the inflammatory mediators in the genital tract of RM in this initial study.

## Methods

### Ethics Statement

The rhesus macaques (*Macaca mulatta*) used in this study were from the California Regional Primate Research Center and they were housed in accordance with the recommendations of the Association for Assessment and Accreditation of Laboratory Animal Care International Standards and with the recommendations in the Guide for the Care and Use of Laboratory Animals of the National Institutes of Health. The Institutional Animal Use and Care Committee of the University of California, Davis, approved these experiments (Protocol # 15835). When immobilization was necessary, the animals were injected intramuscularly with 10 mg/kg of Ketamine HCl (Parke-Davis, Morris Plains N.J.). All efforts were made to minimize suffering. Details of animal welfare and steps taken to ameliorate suffering were in accordance with the recommendations of the Weatherall report, “The use of non-human primates in research”. Animals were housed in an air-conditioned facility with an ambient temperature of 21–25°C, a relative humidity of 40%–60% and a 12 h light/dark cycle. Animals were individually housed in suspended stainless steel wire-bottomed cages and provided with a commercial primate diet. Fresh fruit was provided once daily and water was freely available at all times.

### Animals

The 36 animals used in this study were captive-bred, parous, cycling female rhesus macaques (*Macaca mulatta*) from the California Regional Primate Research Center.

### Sample Collection

Cervicovaginal secretions (CVS) were collected by vigorously infusing 6 ml of sterile PBS into the vaginal canal and aspirating as much of the instilled volume as possible. Care was taken to insure that the cervical mucus was included in the lavage fluid and that no trauma to the mucosa occurred during the procedure. One half of the CVS sample was snap frozen on dry ice and stored at −80°C until analysis. The remainder was spun and the resulting cell pellet was used RNA isolation. The supernatant was treated with 10× Protease Inhibitor (Roche) and subsequently used for cytokine and chemokine quantitation. The sample collection and preparation procedure resulted in at least a 10-fold dilution of the CVS. The menstrual cycles were assessed on the basis of menstrual bleeding, with the first day of menses designated day 0. All CVS sample were collected between day 10 and day 20 of the menstrual cycle.

### Amplification of Cytokine, and Interferon-stimulated Genes by Reverse Transcriptase Real-time PCR

Total RNA was isolated from CVS samples using Trizol (Invitrogen, Grand Island, NY) according to the manufacturer’s protocol. All samples were DNase-treated with DNA-free (Ambion) for 1 hr. at 37°C. cDNA was prepared using random primers and Superscript II (both from Invitrogen). Real-Time PCR was performed using the ABI 7900 Real-Time PCR System (Applied Biosystems, Foster City, CA) as previously described [23]–[25]. Briefly, samples were tested in duplicate and the PCR for the housekeeping gene GAPDH and the target gene were run in parallel on the same plate. The PCR reaction was carried out on a 96 well Optical Plate (Applied Biosystems) in a 25 µl reaction volume containing 5 µl cDNA +20 µl Mastermix (Applied Biosystems). All PCR reactions were run on using the default amplification program: 2 min. at 50°C, 10 min. at 95°C, followed by 45 cycles of 15 s at 95°C and 1 min. at 60°C. Results were analyzed with the SDS 7900 system software, version 2.1 (Applied Biosystems). The mRNA expression levels were calculated from normalized delta Ct (ΔCt) values. Ct values correspond to the cycle number at which the fluorescence due to enrichment of the PCR product reaches significant levels above the background fluorescence (threshold). In this analysis, the Ct value for the housekeeping gene (GAPDH) is subtracted from the Ct value of the target gene. For vaginal lavage samples, the target cytokine mRNA levels in a sample are expressed as the fold increase relative to the GAPDH mRNA levels in the same sample. Also note that amount of RNA extracted from some samples was insufficient for analysis of every host target gene.

### Primer/probe

Sequences for PKR, RIG-I, IL-17, VISA 2′,5′ oligoadenylate synthetase (OAS), Mx, interferon-gamma-inducible protein-10 (IP-10; CXCL10), TNF-α, IL-6, IL-12, monokine-induced by gamma (MIG), MIP-1α, MIP-1b and IFN-gamma have been published previously [23]–[26]. The primer/probe sequences for IFN-alpha were based on the human IFN-alpha 2 gene, Genbank accession number Y11834 [26]. These genes were selected because innate immune responses to bacteria through TLR2 induce the expression of interferons and interferon-stimulated gene products or because they are prototypical mediators of inflammation.

### Quantitation of Cytokines and Chemokines in CVS

The concentration of the inflammatory mediators IL12p70, TNF-α, IL-10, IL-6, IL-1b, IL-8, CXCL10, CXCL8, CCL5, CXCL9, CCL2 in CVS samples collected at Time point 2 were determined using commercial flow cytometric bead array inflammatory cytokine and chemokine kits (BD Bioscience, San Jose, CA) designed for use with human samples. All samples were tested in duplicates, and data were analyzed using FCAP array software (BD Bioscience, San Jose, CA). Note that the volume of some CVS samples was insufficient for analysis of every cytokine/chemokine.

### Sample Processing and Multitag Pyrosequencing to Characterize the Vaginal Microbioata

The methods for DNA isolation and multitag pyrosequencing have been previously described [21], [22]. Briefly, bar-coded primer sets each containing the 27F and 355R 16S rRNA gene primers were used. On the first run with 29 macaque samples, the average number of sequences per sample was 3968 (range 1253–6490) while on the second run with 35 samples, the average number of sequences per sample was 3392 (range 1140–6901). Only forward reads were used to identify bacteria using the Bayesian Classifier provided by the Ribosomal Database II Project (RDP 10). The volume of some CVS samples was insufficient for conducting this analysis.

### Statistical Analysis

The microbiome features, cytokines and chemokines were correlated using a Spearman’s correlation function and then filtered for correlations >0.70 and p<0.05. These correlates were calculated using a custom R module and the correlations and corresponding attributes were imported into Cytoscape [27] for visualization of the network models. The Intersection of the networks was done using the advanced network merge function in Cytoscape.

## Results

### The mRNA of Many Inflammatory Mediators is Readily Detectable in Cervicovaginal Secretions of most RM

**Figure 1:**
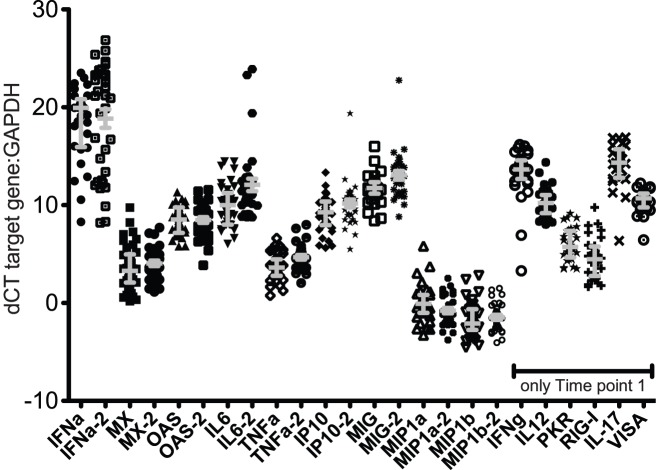
Concentration of all mRNAs (relative to GAPDH) in vaginal secretions collected between menstrual cycle days 10–20 from 36 RM at Time point 1 (March 2011) and from 30–35 RM at Time point 2 (November 2011). The samples collected at Time point 2 are denoted by the notation “−2”. All vaginal secretions were collected between menstrual cycle days 10–20. Note that there was not enough CVS sample at Time point 2 to assess all mRNA targets that were tested at Time point 1. Grey bars denote median and interquartile range of the values.

**Figure 2:**
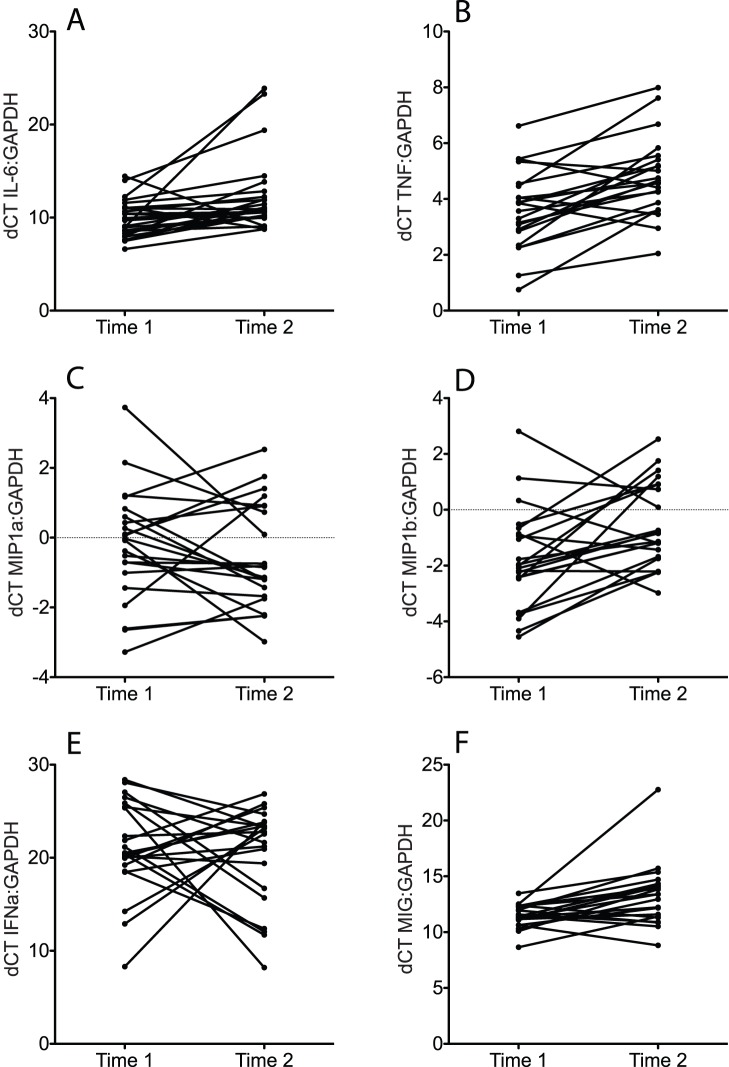
Comparison of relative concentration of for pro-inflammatory cytokine/chemokine mRNAs in vaginal secretions of RM in samples are similar. A) IL-6, B) TNF, C) MIP-1a, D) MIP-1b, E) IFNa, F) MIG. Time 1 indicates the CVS samples were collected in March 2011. Time 2 indicates that the CVS samples were collected in November 2011. All vaginal secretions were collected between menstrual cycle days 10–20.

Of the 15 molecules assessed in the first set of CVS samples collected from 36 rhesus macaques in March 2011 (Time point 1), the mRNA levels of 12 molecules (IFN-α, PKR, RIG-I, IL-17, VISA, OAS CXCL10, TNF, IL-6, IL-12, MIG and IFN-γ) were higher than the GAPDH mRNA levels (dCT>0) in every sample (Figure 1). However, the mRNA levels of MIP-1α and MIP-1b were less than the level of GAPDH mRNA (dCT<0) in most CVS samples (Figure 1). Although the mRNA of most inflammatory molecules tested was elevated, there was a range of 5 - >10 dCT between the samples for all target mRNA (Figure 1). This indicates a wide variation in the expression levels of inflammatory mediators among the animals because a difference of 3 dCT between samples corresponds to a 10 fold difference in mRNA concentration. In the CVS samples collected 8 months later in November 2011 (Time point 2), the mRNA levels of the 9 inflammatory mediators assessed were similar to those found in the Time point 1 CVS samples (Figures 1 and 2). The mRNA levels of proinflammatory mediators (TNF, IL-6, MIP-1α or MIP1b IFNa and MIG) assessed at both time points in 25 animals were compared (Figure 2). Only 2–6 of the 25 animals had a 10-fold difference in the expression levels of TNF, IL-6, MIP-1α or MIP1b IFNa, MIG (Figure 2). Thus, based on mRNA levels of proinflammatory cytokines in CVS, the degree of cervicovaginal inflammation in captive rhesus macaques spans a broad range from minimal to severe but the level of inflammation in an individual animal is stable at least over an 8-month period.

**Figure 3:**
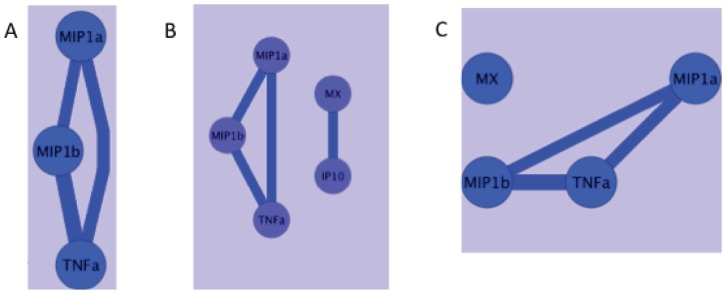
Network of statistical correlations between mRNA levels of immune mediators. After unbiased analysis of potential associations between the levels of every mRNA levels measured using a Spearman’s correlation function there was a limited network of strong (>0.7) correlations between mRNA levels of A) 3 cytokine/chemokines at Time point 1; B) 3 cytokines/chemokines; and 2 Interferon-stimulated genes at Time point 2. C) networks of strong correlations that existed at both Time 1 and Time 2. Blue circles indicate host gene mRNA levels. The lines indicate a positive correlation between the parameters in the circles and the width of the line is proportional to the strength of the correlation.

Correlation network analysis of mRNA levels of the different host genes at Time point 1 (March 2011) showed strong (>0.7 coefficient) positive independent correlations between TNF mRNA levels and MIP1α and MIP1b mRNA levels (Figure 3a). In addition, there was a strong positive correlation between the mRNA levels of MIP1α and MIP1b (Figure 3a). At Time point 2 (November 2011), there were also strong correlations between MIP1α, MIP1b and TNF mRNA levels (Figure 3b). In addition, there was a strong positive correlation between the mRNA levels of Mx and IP-10 at Time point 2 (Figure 3b). The correlations between MIP1α, MIP1b and TNF mRNAs were found at both time points and network analysis demonstrated that these correlations intersect (Figure 3c), thus there was a consistent association between the expression levels of these three inflammatory mediators in the lower female genital tract.

### The Protein Levels of Inflammatory Mediators in Cervicovaginal Secretions Vary Greatly Among RM

**Figure 4:**
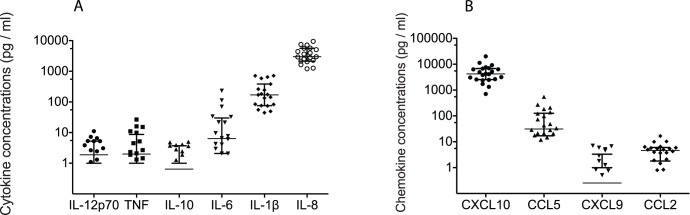
Concentration of A) cytokine and B) chemokine proteins measured in cervicovaginal secretions of RM. All samples were collected between menstrual cycle days 10–20 from 19–22 RM at Time point 2. Bars denote median and interquartile range. Note that if an assay produced a concentration of an analyte below the minimum quantifiable level, a value of zero was assigned and no data points for that sample appears in the graphs.

Of the 12 cytokines and chemokines assessed in the Time point 2 CVS samples collected from 19–22 RM, the median concentration of 3 cytokines IL-6 (median 6.34 pg/ml), IL-1b (median 170.3 pg/ml), IL-8 (median 2997 pg/ml); and 2 chemokines CXCL10 (median 4193 pg/ml), and CCL5 (median 31.21 pg/ml) were higher than 5 pg/ml (Figure 4). The median concentration of IL-12p70 (median 1.88 pg/ml), TNF (median 1.99 pg/ml), IL-10 (median 0.64 pg/ml), CCL2 (median 4.62 pg/ml) and CXCL9 (median 0.26 pg/ml) did not exceed 5 pg/ml in the CVS samples (Figure 4). Although CXCL-10, IL-1b and IL-8 were detected in 100% of samples, CCL2 was detected in 90% of samples, CCL5 was detected in 86% samples, IL-6 was detected in 80% of samples, IL12p70 was detected in 69% of samples, TNF was detected in 65% of samples, IL-10 was detected in 60% of samples and CXCL9 was detected in 50% of samples, Further, there was a wide range (10–1000 fold) in the concentration of every cytokine and chemokine assayed in the CVS samples (Figure 4). This is consistent with wide variation in the levels of genital tract inflammation between the RM in the study. Network analysis of correlations between protein levels of the different host cytokines and chemokines at the second time point showed strong (>0.7 coefficient) positive correlations between IL-8 and IP-10 protein levels and Mx and IP10 mRNA levels. Based in the protein and mRNA levels of inflammatory cytokines and chemokines in the CVS samples, it is apparent that there is extreme variability in the degree of cervicovaginal inflammation between captive rhesus macaques. Further, the mRNA levels of many pro-inflammatory cytokines differed by less than 10 fold at Time point 1 and Time point 2 suggesting that the level of genital inflammation in an animal is relatively stable.

### The Diverse Vaginal Microbiome of RM is Relatively Stable

The lower genital tract microbiota in female rhesus macaques was analyzed at both time points by Multitag pyrosequencing. At Time point 1 (March 2011), 29 macaques were analyzed and the eight most predominant genera were *Porphyromonas, Prevotella, Sneathia, Proteiniphilum, Catonella, Campylobacter, Peptoniphilis* and *Mobiluncus* (Table 1). *Porphyromonas* sequences were present in 93% of the macaques and on average comprised 17% of the sequences.

**Table 1:** Prevalence of Bacteria Genera in Rhesus macaques.

Time 1 (N = 29)			Time 2 (N = 35)		
Genus	% sequences^a^	Freq.^b^	Genus	%sequences	Freq.
Porphyromonas	17	93	Porphyromonas	26	97
Prevotella	14	76	Proteiniphilum	8	54
Sneathia	9	62	Sneathia	8	46
Proteiniphilum	6	69	Mobiluncus	7	77
Catonella	4	41	Prevotella	5	49
Campylobacter	4	83	Atopobium	4	33
Peptoniphilus	4	83	Anaerovorax	4	57
Mobiluncus	3	79	Anaerosphaera	3	60
Anaerovorax	3	52	Catonella	3	34
Ignavigranum	2	34	Soehngenia	3	43
Dialister	2	59	Parvimonas	3	49
Lactobacillus	2	7	Peptoniphilus	2	71
Exilispira	2	28	Gardnerella	2	14
Allisonella	2	38	Lactobacillus	2	9
Anaerosphaera	2	34	Butyricicoccus	1	31

a Average of sequences.

b Percent of macaques with >1% of sequences corresponding to this genus.

Eight months later (November 2011), microbiome from 35 macaques was similarly analyzed. *Porphyromonas* sequences were again present in nearly all (97%) of macaques and were on average the highest fraction of sequences (26%). The other seven most predominant genera were all again present at similar levels except for *Campylobacter*. At both time points the microbiome was relatively diverse with an average of 13 genera at Time point 1 (median 13, range 7–21) and 11.5 genera at Time point 2 (median 11, range 5–20).

*Lactobacillus* was relatively rare in the macaques with only 2% of animals positive at the first time point and 9% at the later time. Analysis of the *Lactobacillus* 16S sequences indicated a close phylogenetic relationship to *L. amylovorus* and *L. johnsonii* (data not shown).

**Figure 5:**
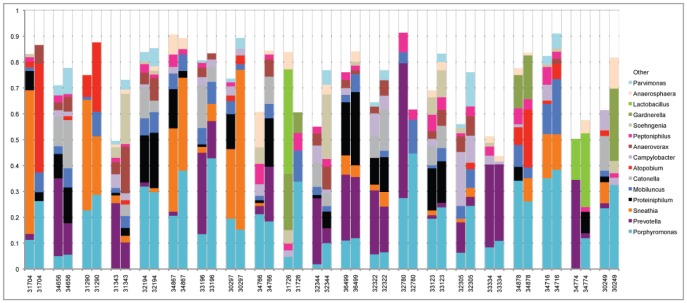
Genera of macaque lower genital tract bacteria. The genital microbiota in 21 macaques was identified at two times (approximately 8 months apart). Each group of two bars represents the relative proportions of 16S sequences indentifying bacterial genera in one macaque at the two different time points. Only the 15 most predominant genera are displayed for clarity.

Twenty-one macaques were analyzed at both time points so that temporal stability of the microbiota could be assessed (Figure 5). While the patterns of microbiota were in some cases very different between macaques, many of the macaques had similar microbiota patterns at the two time points. For example, animal 34656 and animal 31290 each had a distinct pattern of microbiota that was maintained over the two time points; in animal 34656 about 60% of the microbiota was comprised of a combination of *Porphyromonas*, *Prevotella*, *Proteiniphilium*, *Mobiluncus* and *Catonella* but lacked *Sneathia*; while about 60% of the microbiota in animal 31290 was composed of *Porphyromonas* and *Sneathia* but lacked *Prevotella*, *Peptoniphilis*, and *Catonella*. In contrast, microbiota in several of the animals was clearly dissimilar between the two time points (e.g. 34766, 31726). Principal Coordinate Analysis was performed on the 21 sets of microbiota data with two time points to graphically display the similarities and differences in microbiota over time (Figure S1). This analysis showed striking stability in microbiota at the two times for some of the macaques (e.g. 36499, 33123, 32194), moderate to high stability in others (e.g. 34716, 32322) and a few with a large change in microbiota over time (e.g. 31704, 32780).

**Figure 6:**
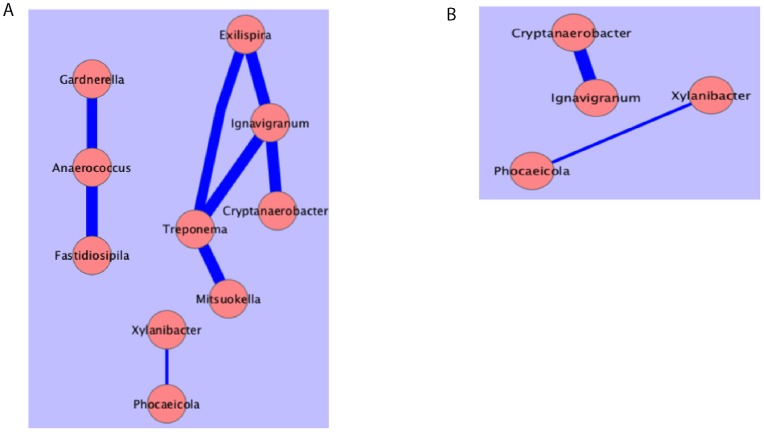
Network of statistical correlations between microbiota. A. Strong (>0.7) correlations between Microbiota at time point 1. B. Intersection of strong correlations that existed at both time 1 and time 2. Pink circles bacterial DNA levels. The blue lines indicate a positive correlation between the parameters in the circles and the width of the line is proportional to the strength of the correlation.

Correlation network analysis between bacteria at the first time point showed strong (>0.7 coefficient) positive correlations of *Anaerococcus* with *Gardnerella* and *Fastidiosipila*. Also, *Ignavigranum* was correlated with three other bacteria; *Treponema*, *Cryptanaerobacter* and *Exlispira* (Figure 6a). A slightly less strong association (>0.5 coefficient) between *Xylanibacter* and *Phocaeicola* was also seen at this time. At the second time point, the strong correlations between *Ignavigranum* and *Cryptanaerobacter* was again observed as well as the association between *Xylanibacter* and *Phocaeicola* (Figure 6b) suggesting very robust associations between these two sets of bacteria. However, the other significant associations between bacteria at Time point 1 were not significant at Time point 2.

### The Relationship between the Vaginal Microbiome and the Levels of Inflammatory Cytokines and Chemokines

**Figure 7:**
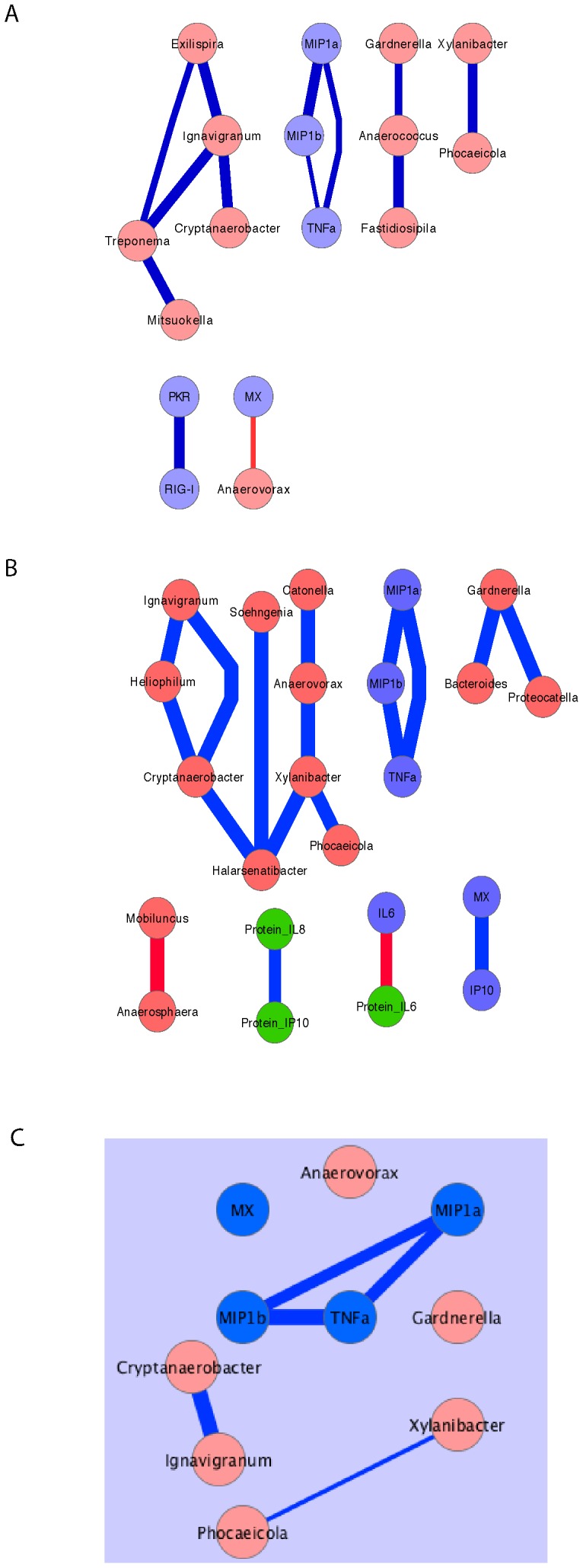
Network of statistical correlations between inflammatory mediators and microbiota. After unbiased analysis of potential associations between host gene mRNA levels and bacterial DNA levels using a Spearman’s correlation function there was a limited network of strong (>0.7) correlations between chemokines/cytokines and microbiota A) at time point 1; B) at time point 2. C) Intersection of strong correlations that existed at both Time 1 and Time 2. Blue circles, host gene mRNA levels, green circles host protein levels, pink circles bacterial DNA levels. The blue lines indicate a positive correlation between the parameters in the circles and the width of the line is proportional to the strength of the correlation. The red lines indicate a negative correlation between the parameters in the circles and the width of the line is proportional to the strength of the correlation.

To determine if differences in microbiota could be influencing cytokine levels in the genital tract, network analysis of microbiota, cytokine protein and cytokine mRNA was performed. These analyses were constrained due to the finding that none of the macaques had a “high lactobacillus” microbiota that corresponded to what in humans is relatively non-inflammatory. In fact, we found very limited associations between pro-inflammatory molecules and microbiota. Although there was a negative correlation between Mx mRNA and *Anaerovorax* (Figure 7a) at Time point 1, this association was not present at Time point 2 (Figure 7b) and thus may not be biologically meaningful. Correlation network analysis also demonstrated that the correlations between MIP1α/b and TNF intersect (Figure 7c) but are not correlated with the presence or absence of specific bacteria. Thus there was a consistent association between the expression levels of these three inflammatory mediators in the lower female genital tract but this inflammation was not correlated with specific microbiota in this set of RM samples.

## Discussion

The levels of genital inflammation influence the efficiency of sexual HIV transmission [1] and HIV acquisition is enhanced by the presence BV [6], [10], [12], [28] [3], [10] The SIV/rhesus macaque system is a well-developed animal model that has been used to study the biology of vaginal HIV transmission, however to date the transmission studies using this model have not taken the levels of preexisting cervicovaginal inflammation into account. In this study, we studied a moderate number of RM and found that some combination of the pro-inflammatory molecules IL-1b, IL-6 and IL-8 was present in the cervicovaginal secretions of all the animals. indicating the presence of cervicovaginal inflammation. However, the concentration of the pro-inflammatory mediators and thus, presumably, the degree of cervicovaginal inflammation varied dramatically among the animals. Some animals had low mRNA and protein levels of inflammatory mediators in CVS while other animals had 100–1000 times higher levels of the same mediators. A recent study documented the levels of 10 cytokines and chemokines in CVS samples collected longitudinally from 30 healthy Caucasian women with genital microbiota dominated by Lactobacillus [29]. Of the 5 molecules that were assessed both in CVS samples from these women and the CVS samples of the RM studied here, the median levels of IL-1b, IP-10 and IL-8 were 10–100 fold higher in RM CVS, the median level of IL-12p70 were 3 fold higher in RM CVS, and the median level of IL-6 was similar in the women and RM. The cytokines/chemokines that were relatively elevated in the CVS of many RM included IL-1b, IP-10 and IL-8 which are classic mediators of inflammation. IL-1b is increased in women with bacterial vaginosis compared to women with a genital microbiota dominated by *Lactobacillus* [9], [10].

In most animals, the mRNA levels of the inflammatory mediators were similar in the 2 CVS samples collected 8 months apart, suggesting that genital inflammation is stable in a subset of captive female RM. It seems likely similar levels of pre-existing cervicovaginal inflammation were present in RM used for published vaginal transmission experiments [30]–[37] and that this influenced the results of these experiments.

The current studies of the RM genital microbiota showed several features that appear to be common to the RM and pigtailed macaques genital microbiota described in recent pyrosequencing studies [21], [22]; 1) the microbiota was relatively diverse especially compared to humans with a “high-lactobacillus” microbiota; 2) there was a low frequency of *Lactobacillus*; 3) when *Lactobacillus* was present, the species were different than those found in humans; and 4) many of the more prevalent genera present in the rhesus macaques are the same as those found frequently in humans with bacterial vaginosis including *Prevotella, Sneathia, Peptoniphilis* and *Mobiluncus*. However, this study showed a notable difference with the previous microbiome studies. Thus, *Porphyromonas* was by far the most predominant genus in these macaques since it was present at fairly high levels in nearly all of the macaques. In contrast, while significant levels of *Porphyromonas* sequences were observed in the two previous studies, [21], [22] the previous rhesus macaque studied had *Sneathia, Mobiluncus and Streptococcus* sequences at the highest levels while the pigtailed macaques had *Sneathia* and *Fusobacterium* sequences at strikingly high levels [21], [22]. Thus, taken together these three studies suggest that the genital microbiota at a primate center can have a characteristic signature pattern. A striking finding was the stability of vaginal microbiota in some of the macaques. Although these animals were sampled 8 months apart, the microbiota in some of the macaques was highly similar at the two time points. However, the microbiota was in most cases very different between animals. A recent study by Gajer et al. [38] shows that microbiota in healthy humans can be relatively stable over a 16-week period, although in most healthy women the genital microbiota was dominated by *Lactobacillus*.

It is worth noting that the protein and mRNA levels for 2 of 3 cytokines tested in both assays did not correlate. However this is not surprising given that the levels of many cytokines including IL-12 and TNF are regulated at the level of post-translation modification and gene expression. Further, the degradation rates of intracellular mRNA and secreted proteins are expected to differ. expected correlations between the mRNA levels of inducer and effector molecules were often in apparent. Thus IFN-a mRNA did not correlate with mRNA levels of the ISGs Mx, OAS and IP-10. Similarly, the mRNA levels of MIG and IFN-gamma in CVS did not correlate despite the fact the IFN-g induces MIG mRNA expression [39]. The lack of correlation in the CVS samples is likely due to the complex mixture of cells, including sloughed mucosal epithelial cells and immune/inflammatory cells) contributing mRNA to the PCR reaction.

The reproductive physiology of female rhesus macaques is complex and could influence the results of the present study. The menstrual cycle length for indoor-housed M. mulatta ranges from 23 through 35 days in the mid-Atlantic and Southeast regions of the U.S.A. [40], [41]. Similarly, rhesus macaques in indoor–outdoor housing in the Chongqing area of China have a menstrual cycle of about 28 days [42]. While menstrual cycles can occur throughout the year in outdoor environments, ovulation in outdoor-housed rhesus macaques is restricted to the fall and winter (mid-Nov though mid-April in the northern hemisphere) [43]. Thus anovulatory menstrual cycles are common in outdoor-housed animals. Rhesus monkeys housed in outdoor, seminatural environments typically exhibit sexual behavior during the fall and winter months when females ovulate [40], [44]. However in indoor laboratory housing, mating and conceptions can occur at any month of the year [40], [41]. Thus, the breeding and ovulatory seasonality found in free-roaming and outdoor housed rhesus macaques is lost as indoor housed animals adapt to the carefully regulated environment. The animals included in this study were housed indoors for at least 2 years prior to sample collection and the CVL samples in the current study were collected in early March and late November. Thus it is unlikely that the reproductive seasonality found in outdoor-housed rhesus macaques influenced the results reported here.

Although the genital microbiota influences the expression of proinflammatory cytokines in women [9], [10], we did not detect a direct association between a specific bacterial genus and the levels of any proinflammatory cytokine. This apparent difference in women and female RM is likely explained by the fact that the normal women in these clinical studies had *Lactobacillius* -dominated vaginal flora, unlike any of the RM in the current study. Thus the current study does not seem to have included any RM that are equivalent to the normal women in these human studies that had no vaginal inflammation. Additional studies that include more RM with little or no vaginal inflammation may help establish a relationship between inflammatory cytokines and vaginal flora. However, the results of this study and the two other recent pyrosequencing studies of genital microbiota in macaques at primate centers indicate that macaques with a genital microbiota that is predominantly *Lactobacillus* is rare and suggests that most macaques have a microbiota that if found in humans would be associated with inflammation. Of note, expression levels of cytokines and ISGs associated with antiviral immune responses, including IFN-alpha, IP-10, MIG, Mx and PKR, were elevated in the CVS of many RM. This response may be due to the presence of an undetected genital viral infection or it may reflect a non-classical response to the vaginal microbiota and future studies should attempt to understand why these antiviral mediators are elevated.

## Supporting Information

Figure S1 Principal Coordinate Analysis of Macaque Microbiota. Each macaque is represented by one type of symbol and there are two points for each macaque, each point representing a separate sampling time. For example, the two points representing the two sampling times for macaque 32194 are closely clustered indicating a high level of relatedness of the bacterial microbiota over time in this animal.
(EPS)
